# Effect and behaviour of different substrates in relation to the formation of aerobic granular sludge

**DOI:** 10.1007/s00253-014-6358-3

**Published:** 2015-01-24

**Authors:** M. Pronk, B. Abbas, S. H. K. Al-zuhairy, R. Kraan, R. Kleerebezem, M. C. M. van Loosdrecht

**Affiliations:** 1 Department of Biotechnology, Delft University of Technology, Julianalaan 67, Delft, 2628 BC The Netherlands; 2 Royal HaskoningDHV B.V., P.O Box 1132, 3800 BC Amersfoort, The Netherlands

**Keywords:** Aerobic granular sludge, Methanol, Alcohol, Aldehyde, Methanogens, Granule formation, Industrial wastewater, Disproportionation, Feeding strategies

## Abstract

When aerobic granular sludge is applied for industrial wastewater treatment, different soluble substrates can be present. For stable granular sludge formation on volatile fatty acids (e.g. acetate), production of storage polymers under anaerobic feeding conditions has been shown to be important. This prevents direct aerobic growth on readily available chemical oxygen demand (COD), which is thought to result in unstable granule formation. Here, we investigate the impact of acetate, methanol, butanol, propanol, propionaldehyde, and valeraldehyde on granular sludge formation at 35 °C. Methanogenic archaea, growing on methanol, were present in the aerobic granular sludge system. Methanol was completely converted to methane and carbon dioxide by the methanogenic archaeum *Methanomethylovorans uponensis* during the 1-h anaerobic feeding period, despite the relative high dissolved oxygen concentration (3.5 mg O_2_ L^−1^) during the subsequent 2-h aeration period. Propionaldehyde and valeraldehyde were fully disproportionated anaerobically into their corresponding carboxylic acids and alcohols. The organic acids produced were converted to storage polymers, while the alcohols (produced and from influent) were absorbed onto the granular sludge matrix and converted aerobically. Our observations show that easy biodegradable substrates not converted anaerobically into storage polymers could lead to unstable granular sludge formation. However, when the easy biodegradable COD is absorbed in the granules and/or when the substrate is converted by relatively slow growing bacteria in the aerobic period, stable granulation can occur.

**Electronic supplementary material:**

The online version of this article (doi:10.1007/s00253-014-6358-3) contains supplementary material, which is available to authorized users.

## Introduction

Aerobic granular sludge (AGS) is an innovative technology to simultaneously remove nitrogen, phosphorus and carbon from wastewater with bacterial granules. This is achieved in one reactor compartment; therefore, the requirement of space and energy is highly reduced (De Kreuk and van Loosdrecht 2006b). The process is operated as a sequencing batch system allowing for optimal process control and flexibility. Full scale aerobic granular reactors treating domestic wastewater are currently operational in The Netherlands, Portugal and South Africa (Giesen et al. 2013). AGS is formed by applying selective environmental pressures on bacteria generally found in sewage treatment sludge (Beun et al. 2000; McSwain et al. 2004). Selection of fast settling over slower settling biomass is commonly reported for the selection of AGS (Jungles et al. 2011; Liu et al. 2005; Lochmatter and Holliger 2014). However, the selection of relative slow growing bacteria is even more or equally important to fully utilize the potential of AGS (de Kreuk et al. 2005b). Easy biodegradable chemical oxygen demand (COD) when supplied under anaerobic conditions can be converted into storage polymers, by organisms such as polyphosphate accumulating organisms (PAOs). The easily degradable COD is in this way stored in a larger fraction of the granule volume. When all soluble COD is removed, the bacteria are supplied with oxygen (and nitrate), and they slowly convert the substrate into CO_2_ and new biomass. This eliminates the presence of fast growing aerobic heterotrophs on dissolved COD and results in smooth dense granules due to growth of heterotrophs throughout the granule (De Kreuk and van Loosdrecht 2004).

As AGS is quickly adopted as technology for the treatment of domestic and some industrial wastewaters, so does the need for further knowledge on the effect of different carbon compounds on the granulation process. As for now, mainly acetate, propionate, ethanol, sugars, molasses and sewage have been used in lab scale reactors to investigate granulation (Beun et al. 1999; Beun et al. 2002b; de Kreuk and van Loosdrecht 2006a; Morgenroth et al. 1997; Tay et al. 2002; Weissbrodt et al. 2013).

The ability of bacteria to store substrates anaerobically plays an important role in the effective formation and stability of AGS. Some substrates might be very difficult or even impossible for bacteria to utilize anaerobically without special requirements, and their impact on the granulation potential and stability is still largely unknown.

In this work, we studied the conversion and evaluated the granulation potential of a set of carbon compounds frequently encountered in industrial wastewaters, i.e. acetate, methanol, butanol, propanol, propionaldehyde and valeraldehyde. Based on the results obtained in this work and those already present in literature, the general effect on AGS of various types of carbon compounds and operational conditions is discussed.

## Material and methods

### Experimental setup

A double walled glass sequencing batch reactor (SBR) with an internal diameter of 6.25 cm, 1.5 m in height and 2.7 L working volume was operated as a bubble column. The temperature of the reactor was controlled at 35 ± 0.5 °C by means of a cryostat similar to the reactor used by De Kreuk et al. (2005c) and Winkler et al. (2011). The influent was preheated to ensure that the reactor remains at the correct temperature during feeding. The off-gas was recirculated with a constant flow of 5 L min^−1^ to keep the dissolved oxygen at its desired set point of 3.5 mg O_2_ L^−1^. The dissolved oxygen concentration in the reactor medium was controlled by supply of a nitrogen gas and air mixture via mass flow controllers. A bio controller (Braun DCU4 coupled with Multi Fermentor Control System acquisition software; Sartorius Stedim Biotech S.A., Melsungen, Germany) was used to control and operate the SBR. The volume exchange ratio was 0.56. The reactor was operated at a cycle length of 3 h, following an anaerobic-aerobic regime as shown in Table 1. The dosage of 1 M NaOH and HCl controlled the pH at 7.1 ± 0.05 during the aeration period. The sludge used for inoculation of the reactor was obtained from an activated sludge treatment plant treating domestic wastewater with phosphorus and nitrogen removal. The total suspended solids (TSS) and volatile suspended solids (VSS) were calculated as described in Pronk et al. (2014).

**Table 1:** Operation of the cycles in the aerobic granular sludge reactor

Phases	Time	Volume
	[min]	[L]
Anaerobic feeding	60	1.5
Aeration	100–112	
Settling	3.–0.15	
Effluent withdrawal	5	1.5
Total cycle length	180	

### Medium

The synthetic medium consisted of 150 mL medium A and 150 mL medium B dosed together with 1200 mL heated tap water, achieving an influent temperature of 35 °C. The composition of medium A consisted of 1.13 g L^−1^ NH_4_Cl, 0.13 g L^−1^ K_2_HPO_4_, 0.05 g L^−1^ KH_2_PO_4_, 0.88 g L^−1^ MgSO_4_·7H_2_O, 0.35 g L^−1^ KCl and 90 mL L^−1^ trace element solution with the following composition: 63.7 g L^−1^ C_10_H_14_N_2_Na_2_O_8_·2H_2_O (EDTA TITRIPLEX® III), 4.99 g L^−1^ FeSO_4_·7H_2_O, 2.2 g L^−1^ ZnSO_4_·7H_2_O, 7.34 g L^−1^ CaCl_2_·2H_2_O, 5.06 g L^−1^ MnCL_2_·4H_2_O, 1.51 g L^−1^ Na_2_MoO_4_·2H_2_O, 1.57 g L^−1^ CuSO_4_·5H_2_O, 3.22 g L^−1^ CoCl·6H_2_O.

The composition of medium B was changed after 64 days of operation. First, medium B consisted of the following: 4.87 g L^−1^ HAc, 0.5 g L^−1^ MeOH, 0.53 g L^−1^PrOH, 0.29 g L^−1^BuOH. After 63 days, the HAc contribution to the COD was halved and substituted by propionaldehyde and valeraldehyde. This changed the concentration of HAc to 2.4 g L^−1^, propionaldehyde to 0.27 g L^−1^ and valeraldehyde to 0.22 g L^−1^ in medium B. The total COD concentration of the influent fed to the reactor was 509 mg COD L^−1^, and the total nitrogen was 29.5 mg N L^−1^.

### Analytical procedures

The COD, ammonia, nitrite, nitrate and phosphate concentrations in the bulk liquid were measured weekly with a spectrophotometer cuvette system from Hach Lange (DR2800). Acetate, methanol, propanol, pentanol, propionaldehyde, butanol, propionic acid, valeric acid and valeraldehyde were quantified by gas chromatography (GC) (Oudshoorn et al. 2009).

Methane concentrations from the anaerobic batch tests were determined with a Varian 3800 custom solution Gas Chromatograph. Gas samples were injected with a 100-μL gastight Hamilton syringe in a Varian Ultimetal 1079 split/splitless, which was operated at 200 °C at a split ratio of 100. A CP-Sil-5CB (50 m × 0.32 mm) capillary column was used isothermally at 100 °C at a constant gas flow rate of 10 mL min^−1^. The used carrier gas was helium. Methane peaks were detected with a Varian Flame Ionisation Detector, which was operated at 300 °C. The helium make-up flow was 25 mL min^−1^, hydrogen flow was 30 mL min^−1^ and the airflow was 300 mL min^−1^. Carbon dioxide samples were separated on a Hayesep Q 80/100 mesh 0.25 × 1/16″ × 1 mm Ultimetal micro packed column via a Varian 1041 on-column direct injector. Helium was applied as carrier gas at a pressure of 12.5 PSI. Temperatures of the TCD detector, column and injector used were 300, 50 and 120 °C, respectively.

Pottered and sliced granules were examined with a Zeiss Axioplan 2 epifluorescence microscope equipped with filter set 06 (bp 436/10 FT 460). To visualize methanogens in these samples, the fluorescence of coenzyme F_420_, present in most methanogens, was used as described by Reuter et al. (1986).

### Analysis of polyhydroxyalkanoates

Biomass samples were collected in 15-mL plastic falcon tubes and freeze-dried. Approximately 40 mg of homogenized freeze-dried sample, 2 mg of mixed standard (88 % PHB, 12 % PHV) and 2 mg of 2-hydroxyhexanoic acid 98 %, 2 mL of chloroform and 2 mL of acidified methanol were put in borosilicate glass tubes and mixed vigorously. The closed tubes were heated for 1 day at 95–100 °C in a heating block. After which, the tubes were cooled down to 4 °C for 30 min. One millilitre of aqueous ammonia solution (14 %) was added and vigorously mixed before centrifugation with 2500 rpm for 5 min. The samples were stored at 4 °C for 1-h to prevent methanol evaporation. About 1.5 mL of the chloroform phase of each tube was collected and inserted into closed GC ampules. One microlitre of the chloroform phase was injected in an Agilent 6890 N gas chromatograph. The chromatograph was operated with an HP-innowax Column (60 m × 0.25 mm × 0.15 μm), helium as a carrier gas (1.7 mL min^−1^). The flame ionization detector (FID) unit was operated at 300 °C with an injection port temperature of 250 °C. The oven temperature was set to 80 °C for 1 min, increased at 10 °C min^−1^ to 120 °C, and then to 270 °C at 45 °C min^−1^ and held for 3 min. The column used was a ZB-Wax (20 m × 0, 18 mm × 0, 18 μm) with helium as a carrier gas (230 kPa). Injector temperature was set at 240 °C; transfer line temperature was 250 °C, the split ratio 1:8. Electron impact ionization was set to 70 eV with a source temperature of 250 °C.

### Off-gas measurements

Gas analysis of the recycle flow was measured online with a Rosemount analytical NGA 2000 MLT gas analyser for carbon dioxide and oxygen. Methane was measured with a Servomex 4900 infrared gas analyser. Both analysers were calibrated regularly with the corresponding gasses. The built-in pressure sensor corrected automatically for changes in atmospheric pressure.

### Batch experiments

Anaerobic batch experiments for the different substrates were performed with OxiTop® Control AN6/AN12. The measuring heads were equipped with a pressure sensor (−360, +360 hPa). Every individual bottle used was carefully calibrated for its volume; the increase of the pressure in the headspace (hPa) could then be related to the conversion of the added substrate via the ideal gas law.

About 0.6 g VSS of granular sludge was added per bottle and filled with influent from the reactor (without the carbon sources) together with a 25 mM HEPES buffer solution set to pH 7.2 (purged with nitrogen gas for 5 min) to a total volume of 200 mL. The bottles were then incubated on a shaker at 190 rpm and at 35 °C. After reaching the required temperature, the elevated pressure (due to the temperature increase) was released with a water lock to maximize the available pressure range. Recording was started and 50 mg MeOH, PrOH, BuOH, PeOH, propionaldehyde, valeraldehyde and 120 mg acetate was injected. Soda lime pellets present in the headspace were used to absorb carbon dioxide in some experiments. An Oxitop® OC110 remote controller was used to monitor and gather the data without the need to disturb the measurement. Batch tests performed with granules from the reactor are summarized in Table 2.

**Table 2:** Anaerobic batch tests with aerobic granules

Substrate	CH_4_	CO_2_	PHA	PrOH	PeOH
Product					
Methanol	+	+	–	–	–
Acetate	–	+	+	–	–
Propanol	–	–	–	–	–
Butanol	–	–	–	–	–
Pentanol	–	–	–	–	–
H_2_/CO_2_	–	–	–	–	–
Propionaldehyde	–	+	+	+	–
Valeraldehyde	–	+	+	–	+

### Sample collection

Granules were taken from the reactor and pottered to create a cell suspension. The cell suspension was washed two times with PBS buffer. The supernatant was discarded, and the pellet was stored at −80 °C.

### DNA extraction

The pelleted cell suspension was extracted after a pre-treatment of grinding under liquid nitrogen, which was repeated three times followed by the usage of the Ultraclean Microbial DNA extraction kit (Mobio, USA) according to the supplied protocol. After extraction, 5 μL of a total of 50 μL of gDNA solution was subjected to gel electrophoresis to check for quality and quantity.

### PCR and DGGE analysis

For the universal detection of the 16 s-rRNA gene from the archaeal domain, we used the following PCR primer set, Parch519fm (Øvreås et al. 1997) in combination with a modified primer Arc934r (5′-GTGCTCCCCCGCCAA-3′) originating from the probe Arc915r developed by Stahl and Amann (1991) which is more specific in the detection of only archaea. For DGGE analysis, a GC-clamp (Muyzer et al. 1993) was added to the 5′-end of the Arc934r primer. For amplification, the following temperature program was used, an initial denaturation of 5 min at 95 °C followed by 28 cycles of 30 s at 95 °C, 40 s at 62.5 °C, 30 s at 72 °C and a final elongation of 30 min at 72 °C. The product (250 ng) was subsequently analysed on DGGE according to Bassin et al. (2011) with the exception that we used a modified Urea-Formamide gradient, 30–60 % and a running protocol of 5 h at 200 V. As an alternative, the near full 16 s-rRNA genes from archaea were amplified using the primers S-D-Arch-0025-a-S17 and S-*-Univ-1517-a-A-21 as describes by Vetriani et al. (1999) and subjected to DGGE analysis. We used a different PCR annealing temperature of 57 °C instead of 48 °C and an elongation time of 90 s at 72 °C. This analysis was performed to confirm the results of the partial 16 s-rRNA gene DGGE.

## Results

### Description of start-up

**Fig. 1:**
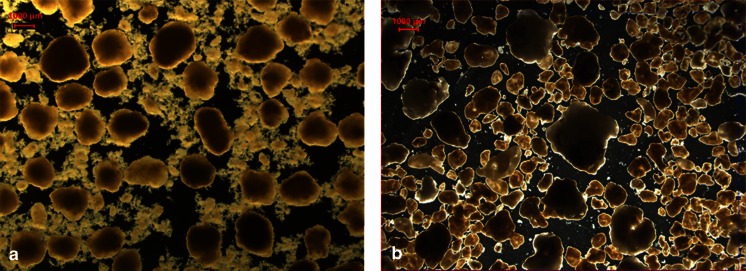
Stereoscopic view of aerobic granules (**a**) day 35 grown on acetate, methanol, propanol and butanol, (**b**) day 225 grown on acetate, methanol, propanol, butanol, propionaldehyde and valeraldehyde; *size bar* is equivalent to 1000 μm

**Fig. 2:**
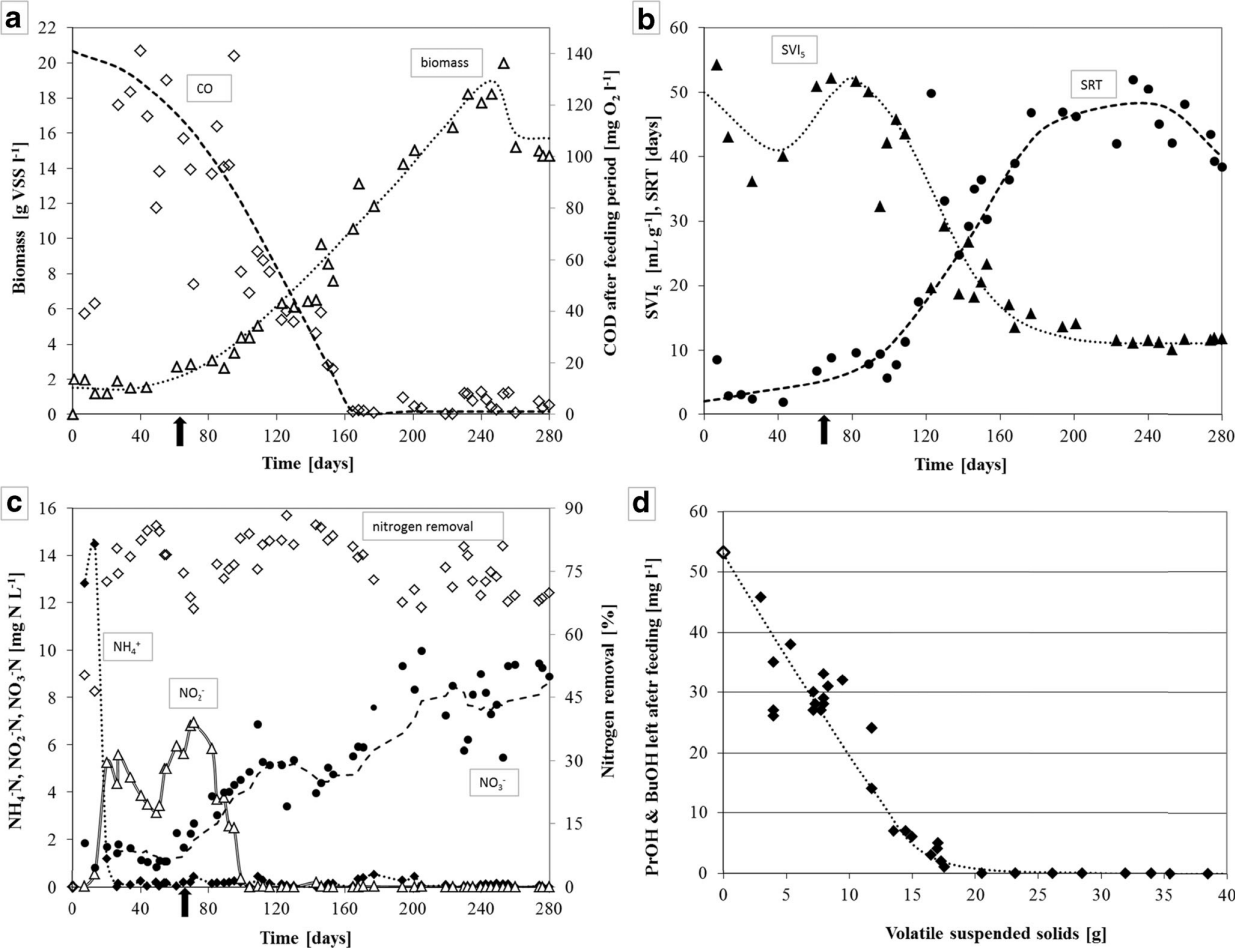
Evolution of biomass growth; (**a**) volatile suspended solid concentration in the reactor (*open triangles*) and chemical oxygen demand in the reactor after the feeding period (*open diamonds*). Evolution of granular sludge; (**b**) sludge volume index (*closed triangles*) and solid retention time (*filled circles*). Nitrogen conversions; (**c**) total nitrogen removal efficiency (*open diamonds*), ammonium (*filled diamonds*), nitrite (*open triangles*) and nitrate (*closed circles*). *Black arrow* indicates the time at which halve of the acetate (COD) was replaced by COD-equivalent amounts of propionaldehyde and valeraldehyde in the medium. *Lines* are shown to indicate trends. Alcohol concentration after the anaerobic feeding; (**d**) decrease of propanol and butanol (*closed diamonds*) found after the anaerobic feeding period versus the volatile suspended solids over time, expected (calculated) concentration of propanol (*open diamond*) in the bulk liquid after the anaerobic feeding period without conversion in a completely mixed reactor

Activated sludge from a conventional wastewater treatment plant in the Netherlands (WWTP Harnaschpolder, Den Hoorn, Netherlands) that had good nitrogen and phosphate removal capability was used to inoculate the reactor. The carbon medium used during the start-up contained the following: acetic acid, propanol, butanol and methanol (see Materials and methods). A settling time of 20 min was selected at first to accommodate the slower settling velocity of the activated sludge and allow the biomass to adapt to the synthetic substrate. This was gradually decreased by 3–5 min over the course of 3 weeks to a final 3-min settling time corresponding to a minimum settling velocity of 12 m h^−1^. The SRT was not actively controlled and was therefore determined by the solids in the effluent. First, granulation occurred after 15 days although flocculent biomass was still predominantly present. Analysis of the bulk liquid after the anaerobic feeding period indicated that roughly 40 % of the COD was not removed by the sludge. The incomplete removal was mainly because methanol, butanol and propanol were initially not taken up during the feeding period. This led to floc formation in the mixed aeration period as can be seen in Fig. 1a. After 64 days, propionaldehyde and valeraldehyde were introduced in the feed replacing a part of the acetate. Total COD was kept constant. Full ammonium removal was already present early in the experimental run, but let to nitrite accumulation (4–6 mg N L^−1^) in the effluent until approximately day 80 (Fig. 2c). The decrease in dissolved oxygen (from 7 to 3.5 mg O_2_ L^−1^) at day 83 resulted in a decrease of nitrite in the effluent due to a higher simultaneous denitrification. During the aeration period, 50 % of the nitrogen was removed via denitrification. Overall nitrogen removal remained roughly 75 % throughout the experiment. Nitrogen removal was not optimized during this experiment. Full removal of the biodegradable COD during the anaerobic feeding period was achieved after approximately 160 days. At this time, the biomass in the reactor had reached approximately 8–9 g VSS L^−1^ (Fig. 2a) and a solid retention time (SRT) of 30 days (Fig. 2b). From this time onward, also the flocculent biomass and the many protozoa previously observed by microscopy disappeared completely from the reactor and the sludge volume index stabilized at a low value (Fig. 2b). At day 256, approximately 100 mL of granules were removed from the reactor for an unrelated experiment, hence the decline in biomass concentration.

### Methanol

During steady operation of the reactor, gas bubbles could be observed escaping from the settled bed during the anaerobic feeding period. Online off-gas measurements showed high concentrations of methane shortly after the aerobic period started. Online quantification of the methane after feeding was found to be troublesome due to strong concentration dynamics in the off-gas. It was therefore difficult to quantify the methane production during the feeding period. In order to quantify methane production during feeding, anaerobic batch experiments were performed. Batch tests with methanol as the only substrate showed that methane was indeed formed. To verify that the methane produced during the anaerobic feeding period was only derived from methanol, also the other carbon compounds present in the feed were evaluated in batch tests. Acetate, propanol, butanol, propionaldehyde and valeraldehyde were not contributing to the production of methane (Table 2). Furthermore, various combinations of the above-described substrates did not yield any methane production, except when methanol was present. A batch test with a gas mixture of hydrogen and carbon dioxide in the headspace did also not yield any methane. The obtained anaerobic methanol conversion rate in batch tests was determined to be 0.4–0.6 m*M* MeOH g VSS^−1^ h^−1^ at day 200. With these conversion rates, only 1.4–2.2 g VSS L^−1^ is required to completely convert methanol during the 1-h feeding period. During the start-up period, methanol was not completely converted during the anaerobic feeding period (Fig. S1). To detect the responsible methanogen species, denaturing gradient gel electrophoresis (DGGE), separating amplified archaeal 16 s rRNA gene fragments, was performed (Figure S2). A single methanogenic archaea with 99.7–99.9 % similarity to *Methanomethylovorans uponensis*, a species out of the *Methanosarcinaceae* family, was found to dominate the aerobic granules. Sequences were deposited into GenBank under accession number KP064473-KP064477.

**Fig. 3:**
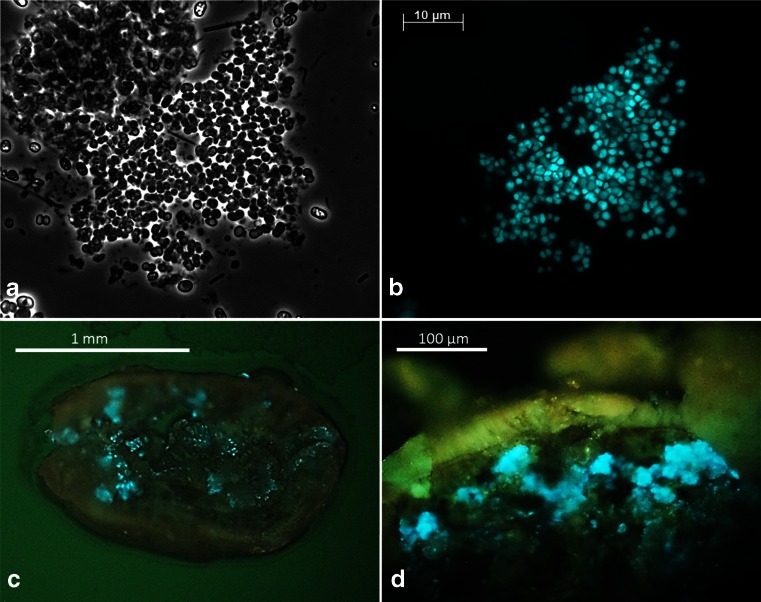
Fluorescence of *Methanomethylovorans uponensis* by excitation of coenzyme F_420_ (exCitation 420, emission 470 nm) in pottered granules; phase contrast (**a**) and fluorescence (**b**) and in a sliced granule (**c**, **d**)

No other methanogenic species were detected. Methanogens in the granules were also detected under ultraviolet light (exCitation at 420, emission 470 nm) using an epifluorescence microscope (Fig. 3). Mainly coenzyme F_420_ present in most methanogens will fluoresce under these conditions, making the presence of methanogens that have this coenzyme easily visible (Reuter et al. 1986). The observed methanogens were growing in large clusters of two to four cells (Fig. 3a, b). They mainly seemed to grow a bit more in the depth of the granule in dense clusters just beneath the surface (Fig. 3c, d).

### Propanol and butanol

Figure 2d indicates that an increase in biomass corresponded with a higher removal of both propanol and butanol during the anaerobic feeding period. Once a significant granular bed (±8–9 g L^−1^) had developed, both alcohols could not be detected anymore after the anaerobic period, and no more flocculent growth was observed (Fig. 1b). This is also reflected by the decrease in COD found after the feeding period (Fig. 2a). At day 64, propionaldehyde and valeraldehyde were introduced in the feed, which led to the release of extra propanol and pentanol from propionaldehyde and valeraldehyde disproportionation, respectively (see below). The extra effective alcohol load was not fully removed in the anaerobic feeding period, and the remaining alcohols in the liquid phase were converted aerobically. This temporarily induced more floc formation and worsened the SVI_5_ of the biomass (Fig. 2b). To further investigate the fate of propanol and butanol during the anaerobic feeding period, a variety of anaerobic batch tests were performed (Table 2). These tests showed no conversion of the alcohols in the storage polymer PHA. Neither were carbon dioxide nor methane produced from these substrates, indicating that no anaerobic bioconversion occurred.

### Aldehydes

**Fig. 4:**
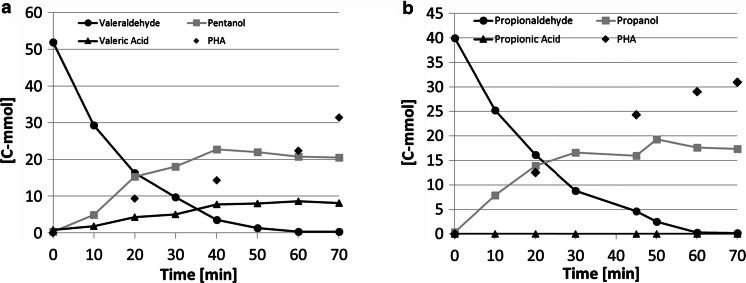
Anaerobic conversion of valeraldehyde (**a**) and propionaldehyde (**b**) at 35 °C, pH 7.2 in batch with granular sludge with 16 and 15 g VSS L^−1^, respectively

Propionaldehyde and valeraldehyde were not detected after the anaerobic feeding period. To investigate the behaviour during the anaerobic feeding, anaerobic barometric batch tests were performed. The measured pressure build-up during the Oxitop batch tests originated from carbon dioxide production (confirmed with GC). Interestingly, propanol and pentanol in the propionaldehyde and valeraldehyde tests, respectively, were detected as being produced. Supplementary batch tests with propionaldehyde and valeraldehyde showed the anaerobic disproportionation reaction of the aldehydes into their corresponding alcohols and carboxylic acids (Fig. 4a, b). Propionic acid produced was completely removed from the liquid by the granules, while valeric acid was only partly removed during the batch tests. Conversions of the aldehydes also lead to PHA accumulation. A carbon balance over the test showed that indeed the conversion of both propionaldehyde and valeraldehyde was almost completely balanced by production of the alcohol, carbonic acid compounds and PHA. The carbon balance closed for 92 and 90 % for propionaldehyde and valeraldehyde, respectively. The theoretical glycogen conversion into PHA has been subtracted, since it was not separately measured (Lopez-Vazquez et al. 2009). Conversion rates derived from batch tests were 1.17 and 0.98 mmol (g VSS h)^−1^ for, respectively, propionaldehyde and valeraldehyde at 35 °C.

## Discussion

### Methane production by aerobic granular sludge

Methanol was completely converted to methane and carbon dioxide during the 1-h anaerobic feeding despite the relative high dissolved oxygen concentration (3.5 mg O_2_ L^−1^) during the 2-h aeration period. The high average SRT of approximately 50 days obtained most likely allowed the growth of *M. uponensis*, which was identified as the only methanogen present in this system. The results from the various carbon sources in the anaerobic batch tests showed that only methanol was converted to methane. This merits the results also found by Cha et al. (2013) with *M. uponensis*. The reported catabolic substrates are trimethylamine, dimethylamine, monomethylamine, methanol, dimethyl sulphide and methanethiol, while H_2_-CO_2_, 2-propanol and acetate are not. Both the reported optimal temperature and pH range of 37 °C and 6.5–7.0, respectively, for *M. uponensis* meet the operational characteristics of the reactor described in this paper.

Utilisation of acetic acid by methanogens is likely prevented in AGS systems by the fast anaerobic acetic acid uptake of phosphate accumulating organisms (PAOs) or glycogen accumulating organisms (GAOs) that are normally present in these systems. In prolonged tests without methanogens present, methanol did not lead to PHA production in the anaerobic period (data not shown). Production of storage polymers from methanol in mixed cultures is often only achieved by nutrient limitation (Dobroth et al. 2011) in aerobic conditions, a situation which will not be met in standard AGS reactors. This absence of bacteria utilising the methanol for the anaerobic storage of PHA is what allowed the methanogens to thrive in this system providing that the SRT is sufficient. Typically, intensely aerated systems are not associated with methanogenic activity as they are strict anaerobes. However, some methanogen species are aero-tolerant (Morozova and Wagner 2007). Perhaps, *M. uponensis* shares this trade. This could explain why methanogens are active in this system where they are potentially exposed to oxygen from time to time. The dense structure of the granule possible also further facilitates the correct environment for methanogens due to the inherent oxygen gradients that are associated with biofilms (Gonenc and Harremoes 1985; Harremoes 1982). Aerobic species on the outer zones of the granules will consume the oxygen, creating oxygen-limiting conditions in the deeper layers. At the end of the cycle when substrates are fully converted, oxygen is expected to be present throughout the granules. Currently, no oxygen inhibition data is available for *M. uponensis*, but due to the presence of a 2-h aerobic period, a certain tolerance or reversible inhibition to oxygen is to be expected.

Our results show that methylotrophic methanogenic archaea can survive in AGS if specific substrates, like methanol, are present in the wastewater. From a wastewater treatment point of view, methane production in AGS is unwanted as it is a potent greenhouse gas. It could potentially lead to explosive situations as methane and oxygen can both be present concurrently in this system. Likely, a lower SRT can be used to prevent methanogens from flourishing when methanol is present in the wastewater. Further experiments are required to investigate possible methods for restricting the proliferation of methanogens in AGS systems, especially if methanol or other similar one-carbon compounds are present.

### Removal of propanol and butanol by aerobic granular sludge

Propanol and butanol did not lead to any significant storage polymer formation during the anaerobic feeding period. In addition, anaerobic batch tests showed no production of CO_2_ or CH_4_ from the alcohols (Table 2). Storage polymer formation from alcohols has been observed, but only in selected strains under nutrient limitation in aerobic conditions and anaerobically with ethanol, albeit very limited (Alderete et al. 1993; Puig et al. 2008). The absence of polymer formation and formation of catabolic products such as CO_2_ or CH_4_ strongly indicate that there is no conversion of the alcohols under anaerobic conditions, even after considerable adaptation time (280 days).

What was observed was that, with increasing biomass, less propanol and butanol were found after the anaerobic feeding period (Fig. 2d). Since there is no sign of bioconversion during the anaerobic feeding period, and the compounds can only be detected in the liquid at high alcohol to biomass ratios, absorption to the granular sludge matrix seems the most logical process occurring. Future experiments are needed to investigate the absorption mechanism for aliphatic alcohols.

Butanol and propanol, but actually most aliphatic alcohols, are well-known to be able to dissolve in lipids due to their hydrophobic properties, which are determined by their partition coefficient with octanol and water (log P(ow)) (Ly and Longo 2004; McKarns et al. 1997; Rowe et al. 1987). Lipids are a major constituent in the bilayer-water interface of bacterial cell membranes; so, a certain absorption capacity can be expected (Ly and Longo 2004; Weber and De Bont 1996). In fact, Thérien et al. (1984) concluded that the longer the carbon chain of the alcohol, the greater the solubility in lipids, when they investigated the influences of aliphatic alcohols on activated sludge. Possibly, further facilitation of the possible absorption in AGS is the presence of a considerable amount of (approximately 15–25 % *w*/*w*) exopolysaccharides (EPS). This EPS has been found to have hydrophobic properties mainly due to the presence of lipids (Adav and Lee 2008; Artiga et al. 2008; Lin et al. 2010; Zheng et al. 2005). Indeed, other hydrophobic compounds such as fluor-quinolones, nitrobenzene and malachite green have been observed to absorb more in AGS than activated sludge (Adav et al. 2008; Amorim et al. 2014; Sun et al. 2008; Zhao et al. 2011).

A beneficial effect of absorption is that both propanol and butanol will be distributed throughout the granule before aeration starts. In the aerobic period, these easy degradable compounds are then converted inside the granules, which prevent deterioration of the granule stability and structure. If easy degradable material diffuses from the liquid to the granules in aerobic conditions, it will be mainly converted in the outer fraction of the granules. This will lead to fluffy outgrowth of the granule surface and less stable granule formation (Van Loosdrecht et al. 1997). This was actually observed in the early stages of the experiment (Fig. 1a) and after an increase in alcohol load due to the addition of the aldehydes (Fig. 2b). In both instances, alcohols remained in the liquid after the anaerobic period, and more instable granule formation was observed. By this absorption mechanism, easily biodegradable substrates that are not converted into storage polymers during the anaerobic period will not lead to granule instability or fluffy outgrowth (Fig. 1b).

### Disproportionation of aldehydes

Propionaldehyde and valeraldehyde were disproportionated into their corresponding carboxylic acids and alcohols during the anaerobic feeding period. In turn, the alcohols and acids produced were absorbed and converted into storage polymers, respectively (Fig. 4a, b). Disproportionation of aldehydes by various dehydrogenases has been reported in yeast and bacteria alike (Mee et al. 2005; Thielen and Ciriacy 1991). The biological treatment of aldehydes is possible in both anaerobic and aerobic systems (Eiroa et al. 2005; Pereira and Zaiat 2009; Qaderi et al. 2011). In AGS systems, the disproportionation of the aldehydes is not only removing the toxicity anaerobically, but also prevents acidification through conversion of the produced acids to storage polymers. Consequently, the transfer of easy biodegradable substrates to the aeration period is limited, and fast heterotrophic growth is reduced. Stable granule formation is therefore expected.

### Influence of substrates and feeding strategy on granular morphology

**Fig. 5:**
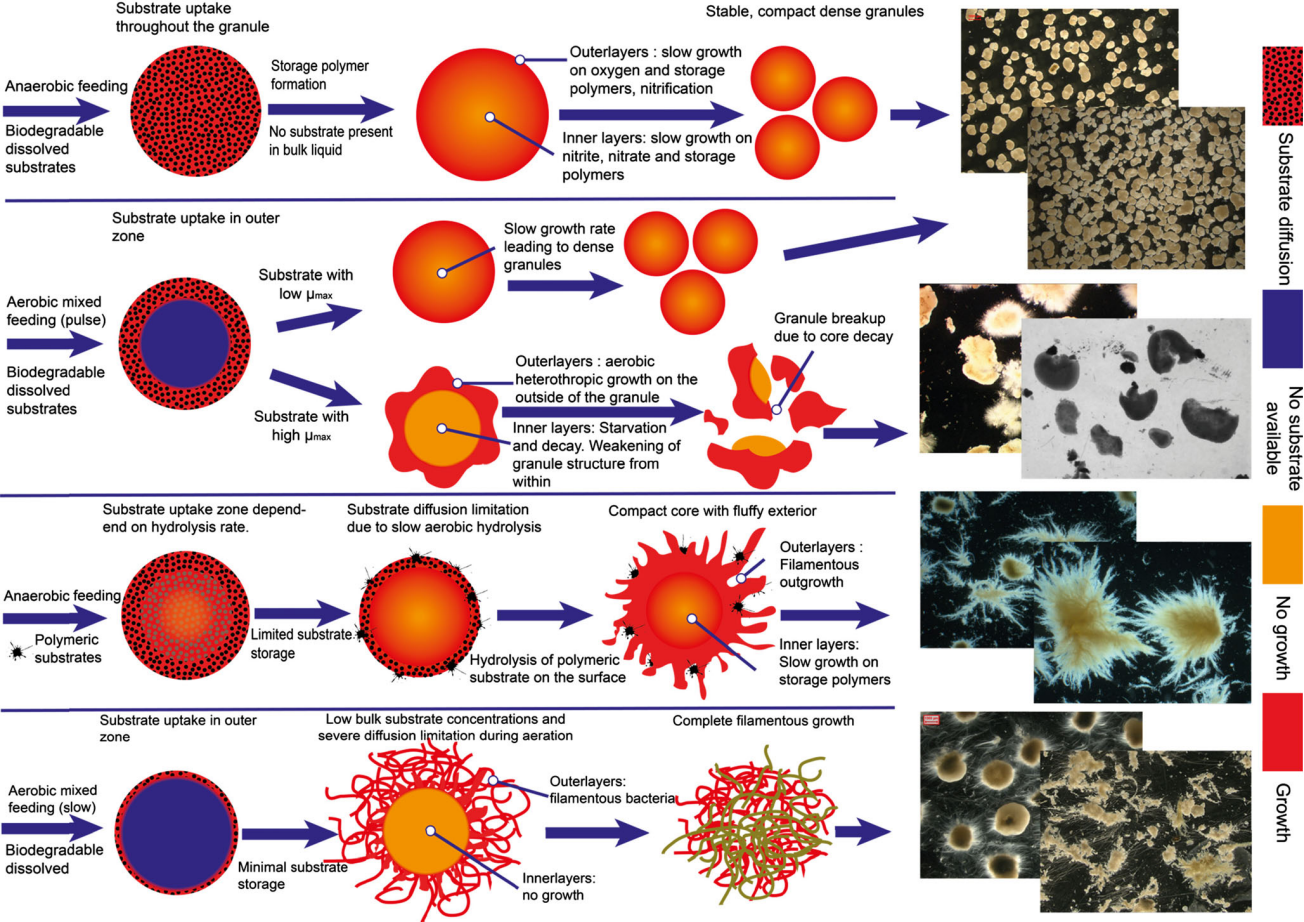
Effect on aerobic granule formation of different carbon sources and feeding regimes in sequencing batch reactors selecting for aerobic granular sludge; biodegradable dissolved substrates fed anaerobically (**a**), aerobic feeding of biodegradable dissolved substrates into a mixed reactor (**b**), anaerobic feeding of polymeric substrates through a settled granular bed (**c**), slow aerobic feeding of biodegradable dissolved substrates in a mixed reactor (**d**)

Wastewater is in general composed of a mixture of substrates. Feeding regimes of AGS bioreactors may vary widely. Both will influence the morphology of the aerobic granules and its stability. The basic principle of stable AGS is the selection of slower growing bacteria and distributing the substrates throughout the granule. This will increase the formation of compact biofilms or granular sludge (Van Loosdrecht et al. 1995). Based on the results of this study and existing knowledge from other substrates present in literature, we have summarised the different feeding conditions and their impact on granular sludge morphology in Fig. 5.

A. Easily biodegradable soluble substrates (i.e. acetate) when fed anaerobically are taken up by PAO or GAO type of bacteria and converted in storage polymers. In a subsequent aerobic period, these storage polymers are used for growth at a relatively slow rate (De Kreuk and van Loosdrecht 2004). By ensuring a relatively high substrate concentration (a few milligrams per litre), due to the anaerobic plug flow feeding, distribution throughout the granules is ensured. This leads to biomass production throughout the granule ensuring granule stability. While no oxygen will be present in deeper regions of the granules, the storage polymers can be oxidized by other electron acceptors such as nitrite and nitrate. These will be produced by nitrification on the aerobic outer layer of the granules. Without nitrification, oxygen will penetrate deeper inside the granule.
  Absorption of substrates (such as the alcohols in this study) that are not sequestered by PAO or GAO like organisms will not lead to granule instability or fluffy outgrowth. Due to absorption, the substrate is not present in the bulk liquid anymore and growth will occur throughout the granule. In this respect, alcohols do not induce granule formation, but rather, they do not affect granule formation negatively. This anaerobic feeding strategy not only selects for stable granulation, but also ensures optimal phosphate and nitrogen removal which is important for the treatment of domestic wastewaters (de Kreuk et al. 2005a).
B. Easy biodegradable soluble substrates dosed fast in an aerobic feeding strategy will lead to substrate or oxygen diffusion limitation. The substrate is used for simultaneous growth and formation of storage polymers mainly limited to outside areas of the granule, while the inner regions are deprived of oxygen (Beun et al. 2002a). The fast consumption of easy biodegradable substrates in the presence of oxygen on the outside fraction of the granule will lead to formation of filamentous outgrowth, increased shear is needed to ensure smooth and stable granulation (Beun et al. 1999). Filamentous growth will increase when the dissolved oxygen concentration in the bulk is decreased below saturation levels (Mosquera-Corral et al. 2005). Granules formed under this regime are more prone to breaking under shear stress since the inner regions are inactive and will eventually decay and weaken the granule (Beun et al. 2002a). This will result in unstable granulation and poor settling characteristics (higher SVI) combined with higher suspended solids (flocs and loose cells) in the liquid after fast settling. Besides this, also the nitrification and phosphorus removal potential is decreased. The slow growing nitrifying bacteria will be overgrown and pushed down to oxygen limited layers by the faster growing heterotrophs (Elenter et al. 2007; Gonenc and Harremoes 1990).
  Some substrates also lead to good granulation even if they are converted aerobically. Ammonium and methanol are such substrates. Both these substrates are converted with oxygen by relatively slow growing bacteria, which leads to a denser biofilm formation (Mosquera-Corral et al. 2003; Villaseñor et al. 2000). In AGS systems, substrates that induce slow growth aerobically are therefore generally expected to lead to stable granulation.
C. Particulate substrates (i.e. starch, proteins) present another challenge, because of the need for hydrolytic conversions. Particulate substrates are mainly hydrolysed at the surface of the granules during steady state (de Kreuk et al. 2010). The hydrolysis products will thereafter be converted into storage polymers. Under anaerobic conditions, PAO and GAO like organisms will be selected, and good granulation will occur. Depending on the anaerobic hydrolysis rate, also aerobic hydrolysis will occur. Under aerobic conditions, the hydrolysis product will be directly used for growth by the organisms at the surface of the granules with steep substrate diffusion limitation gradients (Mosquera-Corral et al. 2003). This will induce filamentous outgrowth, less stable granule formation and higher suspended solids in the liquid phase.
D. Easy biodegradable substrates fed slowly in a mixed aerobic environment will lead to severe substrate diffusion limitation gradients. This provides very good conditions for the proliferation of filamentous organisms (Martins et al. 2003; Martins et al. 2011). AGS fed under these conditions will therefore quickly deteriorate. Breakage of the granules will occur as the inside will not receive any substrate and die. Filamentous growth will have detrimental effect on the settling properties of the sludge and thus on the effluent quality. Granulation formation is thus unlikely.

To summarize, easy biodegradable substrates can have different behaviours during the anaerobic period of the AGS process. Volatile fatty acids are converted by PAO and GAO like organisms into storage polymers, methanol can be converted by methylotrophic methanogens to methane, high carbon alcohols (i.e. propanol and butanol) adsorb in the granule, while the aldehydes are disproportionated in an alcohol and a volatile fatty acid. Easy biodegradable substrates not converted into storage polymers would lead to unstable granular sludge formation unless the substrate is absorbed in the granules and/or select for relatively slow growing bacteria in the aerobic period.

## Electronic supplementary material

Below is the link to the electronic supplementary material.

ESM 1 (PDF 197 kb)
