# Supplementary material for: Effect and behaviour of different substrates in relation to the formation of aerobic granular sludge
Source: Appl Microbiol Biotechnol. 2015 Jan 24;99(12):5257–68. doi: 10.1007/s00253-014-6358-3 (PMC4445487; doi:10.1007/s00253-014-6358-3)
Supplement: Supplementary file 1 — (PDF 197 kb) [file 253_2014_6358_MOESM1_ESM.pdf]

EFFECT AND BEHAVIOUR OF DIFFERENT SUBSTRATES IN RELATION TO THE FORMATION OF AEROBIC GRANULAR SLUDGE

M. Pronk,[1]* B. Abbas[1], S.H.K. Al-zuhairy[1], R. Kraan[2], R. Kleerebezem[1], M.C.M van Loosdrecht [1]*

Department of Biotechnology, Delft University of Technology, Delft, The Netherlands[1] and Royal HaskoningDHV B.V., P.O Box 1132, 3800 BC Amersfoort, The Netherlands[2]

* Corresponding author. Mailing address: Delft University of Technology, Department of Biotechnology, Julianalaan 67, Delft, 2628 BC, The Netherlands. Phone: 31152781618. Fax: 31152782355. E-mail: M.pronk@tudelft.nl, M.C.M.vanLoosdrecht@tudelft.nl

Journal: Applied Microbiology and Biotechnology

Figure S1: Evolution over time of alcohols

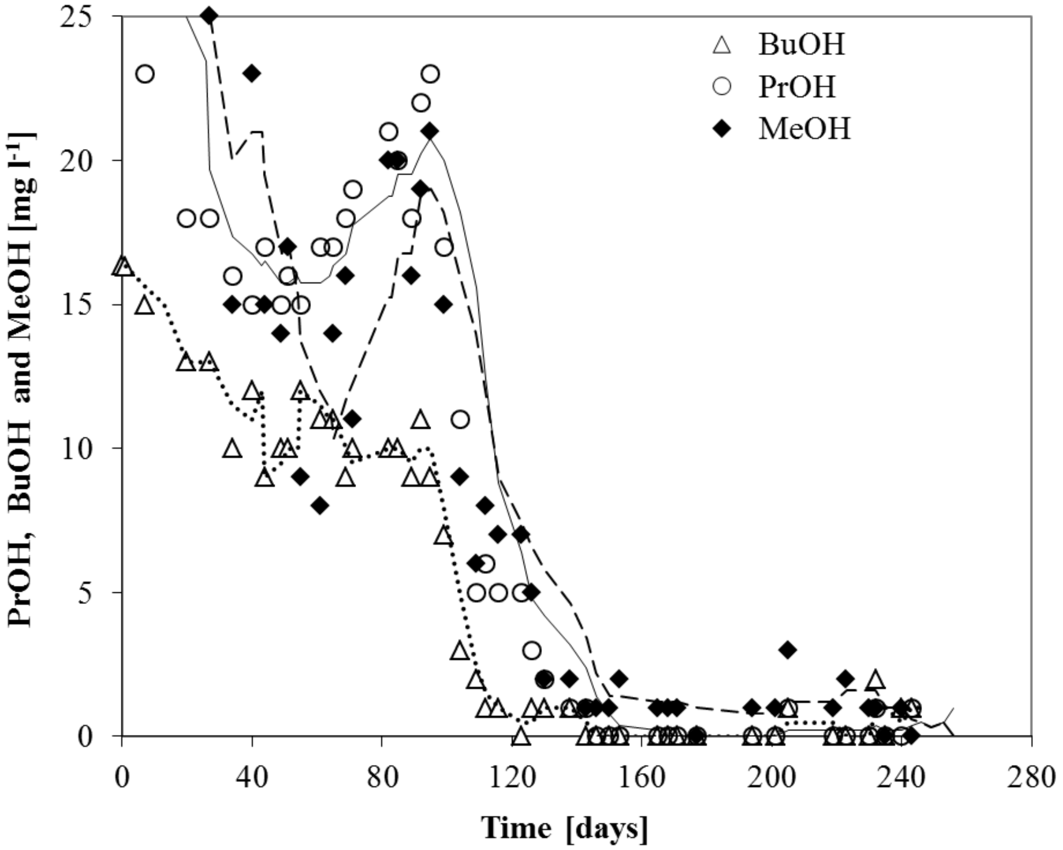

Alcohol concentrations measured in the mixed bulk liquid after the anaerobic feeding period in time.

Figure S2: Denaturing Gradient Gel Electrophoresis of archaeal 16s rDNA gene

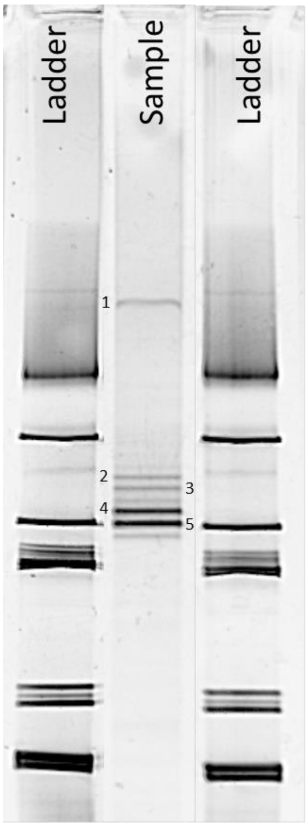

Denaturing Gradient Gel Electrophoresis (DGGE), separating amplified archaeal 16s rDNA gene fragments of granules from the reactor. Numbering 1 through 5 in the above figure are represented in GenBank Accession Number: KP064473-KP064477 by DGGE_B01 – DGGE_B05.
